# Intravenous administration of the selective toll-like receptor 7 agonist DSR-29133 leads to anti-tumor efficacy in murine solid tumor models which can be potentiated by combination with fractionated radiotherapy

**DOI:** 10.18632/oncotarget.7928

**Published:** 2016-03-05

**Authors:** Simon J. Dovedi, Amy L. Adlard, Yosuke Ota, Masashi Murata, Eiji Sugaru, Erina Koga-Yamakawa, Ken Eguchi, Yuko Hirose, Setsuko Yamamoto, Hiroki Umehara, Jamie Honeychurch, Eleanor J. Cheadle, Gareth Hughes, Philip J. Jewsbury, Robert W. Wilkinson, Ian J. Stratford, Timothy M. Illidge

**Affiliations:** ^1^ Targeted Therapy Group, Institute of Cancer Sciences, Manchester Cancer Research Centre, University of Manchester, Manchester Academic Health Sciences Centre, Manchester, UK; ^2^ Manchester Pharmacy School, Manchester Cancer Research Centre, University of Manchester, Manchester, UK; ^3^ Sumitomo Dainippon Pharma, Konohana-ku, Osaka, Japan; ^4^ AstraZeneca Pharmaceuticals Ltd., Alderley Park, Cheshire, UK; ^5^ Current address: MedImmune Ltd., Granta Park, Cambridge, UK

**Keywords:** TLR7, immunotherapy, radiotherapy, radiation, toll-like receptor

## Abstract

Strategies to augment anti-cancer immune responses have recently demonstrated therapeutic utility. To date clinical success has been achieved through targeting co-inhibitory checkpoints such as CTLA-4, PD-1, and PD-L1. However, approaches that target co-activatory pathways are also being actively being developed. Here we report that the novel TLR7-selective agonist DSR-29133 is well tolerated in mice and leads to acute immune activation. Administration of DSR-29133 leads to the induction of IFNα/γ, IP-10, TNFα, IL-1Ra and IL-12p70, and to a reduction in tumor burden in syngeneic models of renal cancer (Renca), metastatic osteosarcoma (LM8) and colorectal cancer (CT26). Moreover, we show that the efficacy of DSR-29133 was significantly improved when administered in combination with low-dose fractionated radiotherapy (RT). Effective combination therapy required weekly administration of DSR-29133 commencing on day 1 of a fractionated RT treatment cycle, whereas no enhancement of radiation response was observed when DSR-29133 was administered at the end of the fractionated RT cycle. Combined therapy resulted in curative responses in a high proportion of mice bearing established CT26 tumors which was dependent on the activity of CD8^+^ T-cells but independent of CD4^+^ T-cells and NK/NKT cells. Moreover, long-term surviving mice originally treated with DSR-29133 and RT were protected by a tumor-specific memory immune response which could prevent tumor growth upon rechallenge. These results demonstrate that DSR-29133 is a potent selective TLR7 agonist that when administered intravenously can induce anti-tumor immune responses that can be further enhanced through combination with low-dose fractionated RT.

## INTRODUCTION

Recent clinical successes with immune-targeting agents demonstrate the potential to augment the anti-cancer immune response through blockade of co-inhibitory pathways. Preclinical and early clinical data also demonstrate the potential for targeting co-activatory immune pathways to facilitate tumor control.

TLRs recognise a diverse repertoire of highly evolutionarily conserved pathogen-associated molecular patterns present on foreign pathogens and are the principle innate differentiators between self and non-self. Signalling through TLR7, which recognises viral RNA motifs, leads to the activation of distinct antigen presenting cells (APCs) such as plasmacytoid dendritic cells (pDCs), expression of type-I and type-II interferon (IFN) and generation of strong T_H_-1 biased immune responses supporting CD8^+^ T-cell and natural killer (NK)-cell mediated immunity [1–3]. Moreover, the induction of type-I IFN has recently been shown to be critical for the generation of competent anti-tumor immune responses [4]. Imiquimod (Aldara, Graceway Pharmaceuticals) is a topically administered TLR7 agonist and is FDA-approved for the treatment of certain forms of dermatologic malignancy [5]. However, the therapeutic efficacy of topically administered TLR7 agonists is generally limited to the treatment site. As such the utility of topically administered agonists may not be optimal for the treatment of non-dermatologic disease.

Two of the key challenges for cancer immunotherapy include increasing the robustness of the anti-cancer response to improve long-term outcome, and increasing the frequency of responders. We and others have demonstrated the potential to augment the efficacy of immunotherapies targeting co-activatory and co-inhibitory pathways by combination with radiotherapy (RT) [6–8]. RT can lead to immunogenic tumor cell death through the release of damage-associated molecular patterns (DAMPs) including High Mobility Group Box 1 (HMGB1), ATP and sensing of cytosolic DNA which can lead to the recruitment and activation of APCs, and priming of tumor antigen-specific T-cell responses [9–14]. In addition to engendering adaptive immunity, RT has also been shown to modulate NK responses through upregulation of NKG2D ligand expression on tumor cells [15] and complement activation [16]. Moreover, RT is an important component of the standard of care for many solid malignancies with 50–60% of all patients receiving this treatment. Therefore RT/immunotherapy combinations are highly relevant for multiple patient populations.

In this study we describe the therapeutic activity of the novel, systemically administered small molecule TLR7-selective agonist DSR-29133. As a monotherapy, DSR-29133 has anti-cancer activity in primary and metastatic mouse syngeneic models. Moreover, we show that the efficacy of DSR-29133 can be augmented by concurrent but not sequential combination with low-dose fractionated RT. Combined therapy leads to a complete response in a high proportion of mice that are protected against disease recurrence by tumor antigen-specific memory immune responses. These studies provide proof of principle for translation to early phase clinical trial.

## RESULTS

### DSR-29133 is a specific TLR7 agonist capable of acute immune stimulation following systemic administration

**Figure 1:**
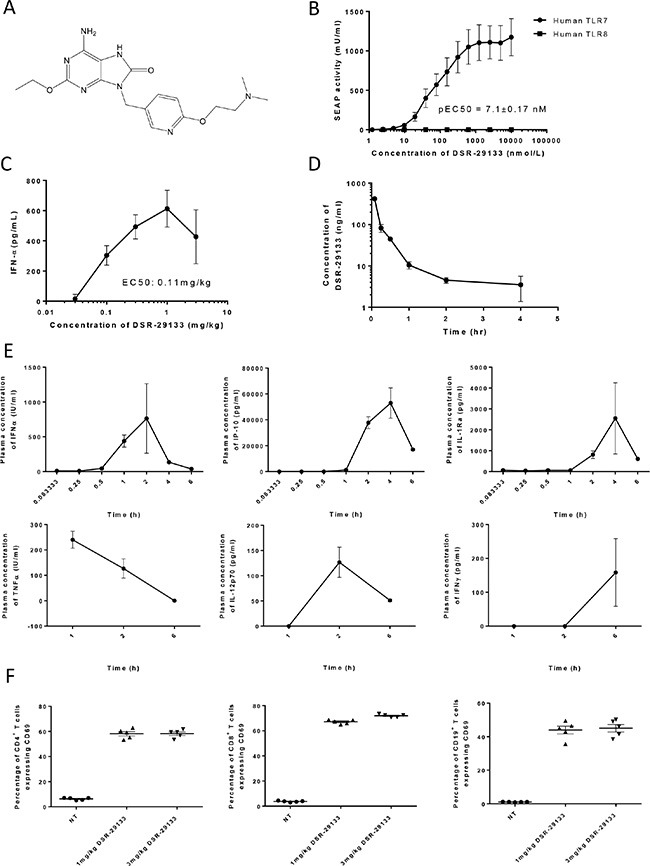
DSR-29133 is a specific TLR7 agonist and induces systemic immune activation following i.v. administration (**A**) Structure of DSR-29133. (**B**) NFκB gene reporter assays were performed in HEK293 cells transfected with either human TLR7 or TLR8 using a range of concentrations (0.001–10 μM) of DSR-29133. (**C**) Plasma IFN**α** concentration was determined after i.v. administration of 0.03–3 mg/kg of DSR-29133. (**D**) Pharmacokinetic profile following i.v. injection of DSR-29133 (at 1 mg/kg) over 4 hours. (**E**) Serum IFN**α**, IP-10 and IL-1Ra were measured 0.08–6 hours and TNF**α**, IL-12p70 and IFNγ 1–6 hours after administration of 1 mg/kg DSR-29133. Data expressed as mean ± S.D. (C) Experimental groups contained 4 Balb/c mice. (D and E) Experimental groups contained 2 mice. Data are representative of at least 2 independent experiments.

This is the first paper describing the novel small molecule TLR7 agonist DSR-29133 (structure defined in Figure 1A). Using NF-kB/SEAP/293 cells stably transfected with either human TLR7 or TLR8 (or mouse TLR7, Supplementary Figure 1) we demonstrate that DSR-29133 can dose-dependently activate TLR7 and is selective against the structurally similar TLR8 (Figure 1B). Assay of plasma IFNα concentration 2 hours after administration of a single i.v. dose of DSR-29133 (0.03–3 mg/kg) determined an EC50 of 0.11 mg/kg (Figure 1C). The maximum plasma concentration (Cmax) of DSR-29133 was achieved 5 minutes after a single i.v. dose of 1 mg/kg DSR-29133 (Figure 1D). Using the same dose of DSR-29133, time-course studies revealed early induction of TNFα and IFNα with the time of maximal plasma concentration (Tmax) occurring 1 and 2 hours respectively after dosing. This was followed by induction of IP-10, IL-1Ra (both with a Tmax of 4 hours) and then by IFNγ (Tmax of 6 hours) (Figure 1E). To confirm activation of immune effector cells, spleens were harvested 6 hours post administration of DSR-29133 (at doses of 1 and 3 mg/kg) and expression of CD69 was determined by flow cytometry (Figure 1F). DSR-29133 significantly induced CD69 expression on CD4^+^ T-cells from 6.3% (CD4^+^CD69^+^ of total CD4^+^) in non-treated control mice to 58.2% (for mice that received either 1 or 3 mg/kg). A similar pattern of response was also observed in CD8^+^ T-cells (from 3.7% to 67.2% and 72% for 1 and 3 mg/kg doses respectively) and CD19^+^ B-cells (from 1.1% to 44.0% and 45.0% for 1 and 3 mg/kg doses respectively).

These data demonstrate that DSR-29133 is a selective TLR7 agonist that can lead to systemic immune activation following i.v. administration.

### DSR-29133 has single agent activity in local and metastatic disease settings

**Figure 2:**
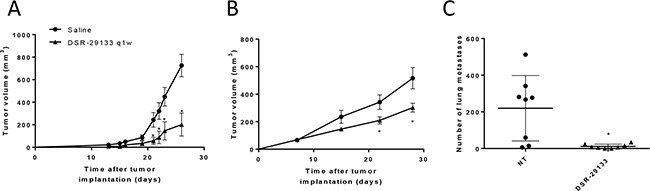
Systemic administration of DSR-29133 1qw improves primary and metastatic tumor control (**A–C**) Mice bearing Renca (A) or LM8 (B and C) tumors received either vehicle (saline) or 0.1 mg/kg DSR-29133 once weekly (1qw) for up to 4 weeks i.v.. (C) Lung metastases were scored by histopathological assessment at the study endpoint (day 28). (A and B) tumor volumes are plotted from the day of therapy (day 0) until the first tumor from each group reached the primary endpoint. Experimental groups contained at least 5 mice and are representative of at least 2 independent experiments. *P < 0.05 (Mann-Whitney test), ^+^, denotes significance (*P* < 0.05; Mann-Whitney test) when compared to monotherapy.

Weekly i.v. administration of DSR-29133 was well tolerated (Supplementary Figure 2) and reduced tumor volume by 72.3% (725.8 ± 99.3 mm^3^ and 201.0 ± 102.2 mm^3^; in control *vs.* DSR-29133 treated respectively at day 26) in the Renca model; and by 41.5% (516.3 ± 77.3 mm^3^ and 302.3 ± 32.1 mm^3^; in control *vs.* DSR-29133 treated respectively at day 28) in the LM8 model (Figure 2A and Figure 2B, Supplementary Figure 3). When inoculated s.c. the osteosarcoma cell line LM8 rapidly metastasizes to the lungs. In addition to improving local tumor control, weekly administration of DSR-29133 significantly reduced the number of pulmonary metastases by 94.6% (219.6 ± 63.14 *vs.* 1.8 ± 4.3; for control and DSR-29133 treated respectively at day 28) (Figure 2C).

These data demonstrate that i.v. administration of DSR-29133 can lead to improved local and disseminated tumor control when administered 1qw for up to 5 cycles as a monotherapy. However, monotherapy with DSR-29133 did not lead to complete rejection in any of the established experimental tumors tested.

### DSR-29133-mediated tumor control can be improved by combination with fractionated RT and leads to expansion of tumor antigen-specific CD8^+^ T-cells

**Figure 3:**
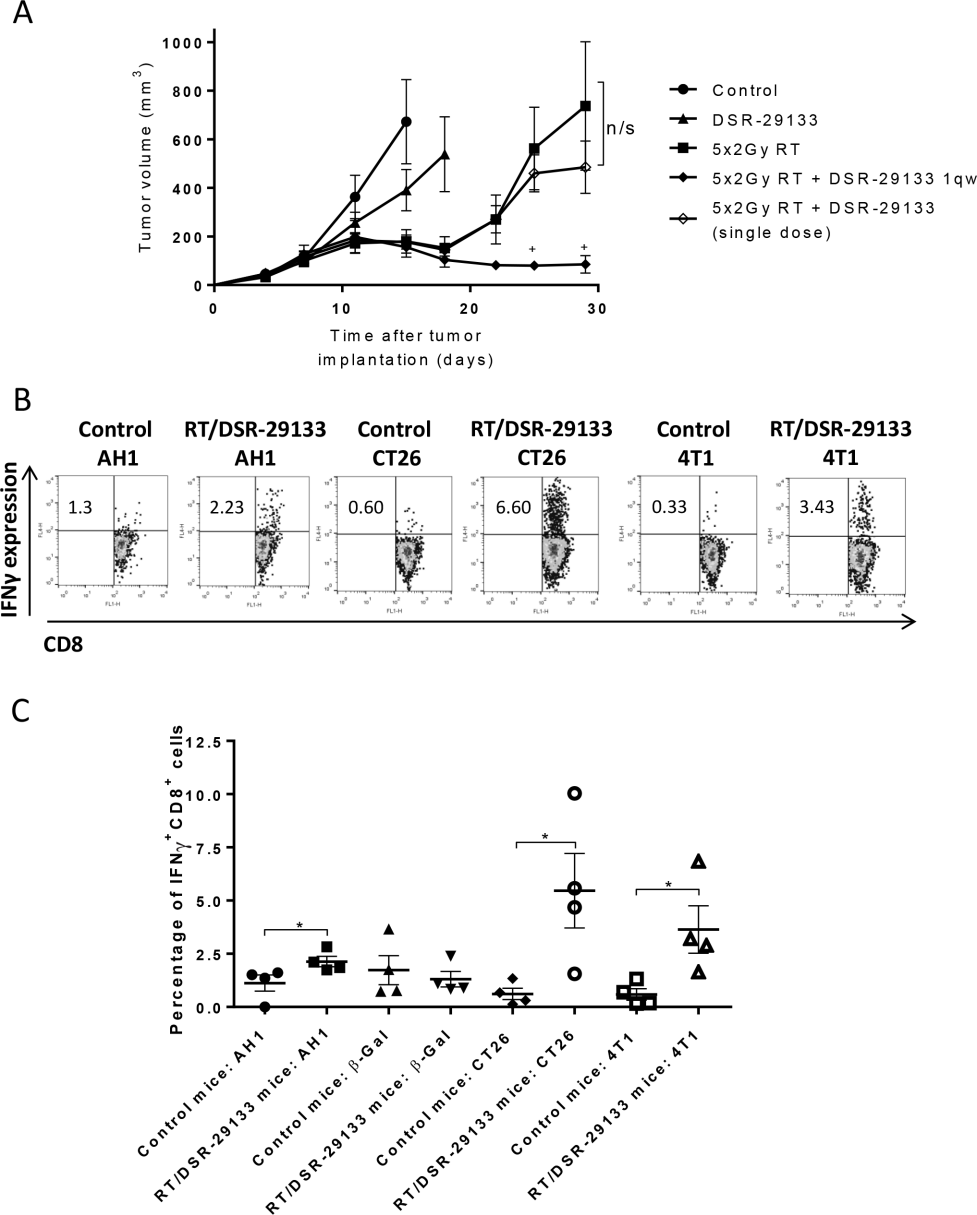
Efficacy of DSR-29133 dosed 1qw can be improved by combination with fractionated RT and leads to expansion of tumor antigen-specific CD8^+^ T-cells (**A**) 10 Gy RT was delivered as daily fractions of 2 Gy over 5 days alone or in combination with either a single i.v. dose, or weekly doses (for up to 4 weeks) of DSR-29133 (at 1 mg/kg). Tumor volumes are plotted from the day of therapy (day 0) until the first tumor from each group reached the primary endpoint. Experimental groups contained at least 7 mice and are representative of at least 2 independent experiments. ^+^, denotes significance (*P* < 0.05; Mann-Whitney test) when compared to monotherapy. (**B**) Representative dot blot of IFNγ production by CD8^+^ T-cells isolated from either control or DSR-29133/RT treated mice. (**C**) Frequency of IFNγ^+^ CD8^+^ T-cells isolated from control or DSR-29133/RT treated mice following co-culture with either H2-Ld restricted peptides (AH1 (SPSYVYHQF); a defined CT26 tumor-associated antigen or β-galactosidase (TPHPARIGL); control peptide of prokaryotic origin), 50 Gy irradiated CT26 or 4T1 cells for 5 days, followed by priming with 50 Gy irradiated tumor cells. *P < 0.05 (Mann-Whitney test). Experimental groups contained at least 4 mice and are representative of at least 2 independent experiments.

In a bid to improve therapeutic response we evaluated the efficacy of DSR-29133 in combination with fractionated RT. Initial studies using colony forming assays determined that DSR-29133 was not directly impacting on tumor cell viability or acting as a radiosensitiser (Supplementary Figure 4A and 4B). *In vivo* efficacy studies in established CT26 tumors demonstrate that local control following DSR-29133 (dosed 1qw for 5 cycles) could be significantly improved when combined with fractionated RT (Figure 3A). In contrast, no improvement in tumor control was observed when RT was delivered in combination with a single dose of agonist, demonstrating the requirement for the 1qw dosing regimen.

To determine whether the combination of DSR-29133 and RT generated tumor antigen-specific T-cell responses, splenocytes were harvested from mice 10 days after the start of combination therapy, and the capacity of CD8^+^ T-cells to produce IFNγ following co-culture with either a gp70 antigenic peptide AH1 (SPSYVYHQF), a control peptide (β-galactosidase: TPHARIGL), irradiated CT26 cells or with irradiated 4T1 cells (which also express the gp70 antigen) was assessed (Figure 3B and 3C). We show that DSR-29133/RT-treated mice have a significantly greater frequency of IFNγ-producing CD8^+^ T-cells following co-culture with AH1 peptide than control mice (2.13 ± 0.25% vs. 1.12 ± 0.38% respectively; *P* < 0.05, Mann-Whitney test). In contrast, no significant difference in CD8^+^ T-cell populations between DSR-29133/RT-treated and control mice was observed following co-culture with the control peptide. When co-cultured with irradiated CT26 cells, DSR-29133/RT-treated mice were endowed with ∼9 fold more IFNγ-producing CD8^+^ than controls (5.47 ± 1.75% vs. 0.61 ± 0.27% respectively; *P* < 0.05, Mann-Whitney test). Moreover, DSR-29133/RT-treated mice also demonstrated an increased frequency of CD8^+^ T-cells capable of recognizing 4T1 cells than control mice (from 3.64 ± 1.12% vs. 0.59 ± 0.27% respectively; *P* < 0.05, Mann-Whitney test).

These data demonstrate that tumor control mediated by DSR-29133 dosed 1qw can be improved when delivered in combination with fractionated RT and lead to the generation of systemic tumor antigen-specific T-cell responses.

### Tumor control following combination therapy with DSR-29133 and fractionated RT is CD8^+^ T-cell dependent and generates immunological memory

**Figure 4:**
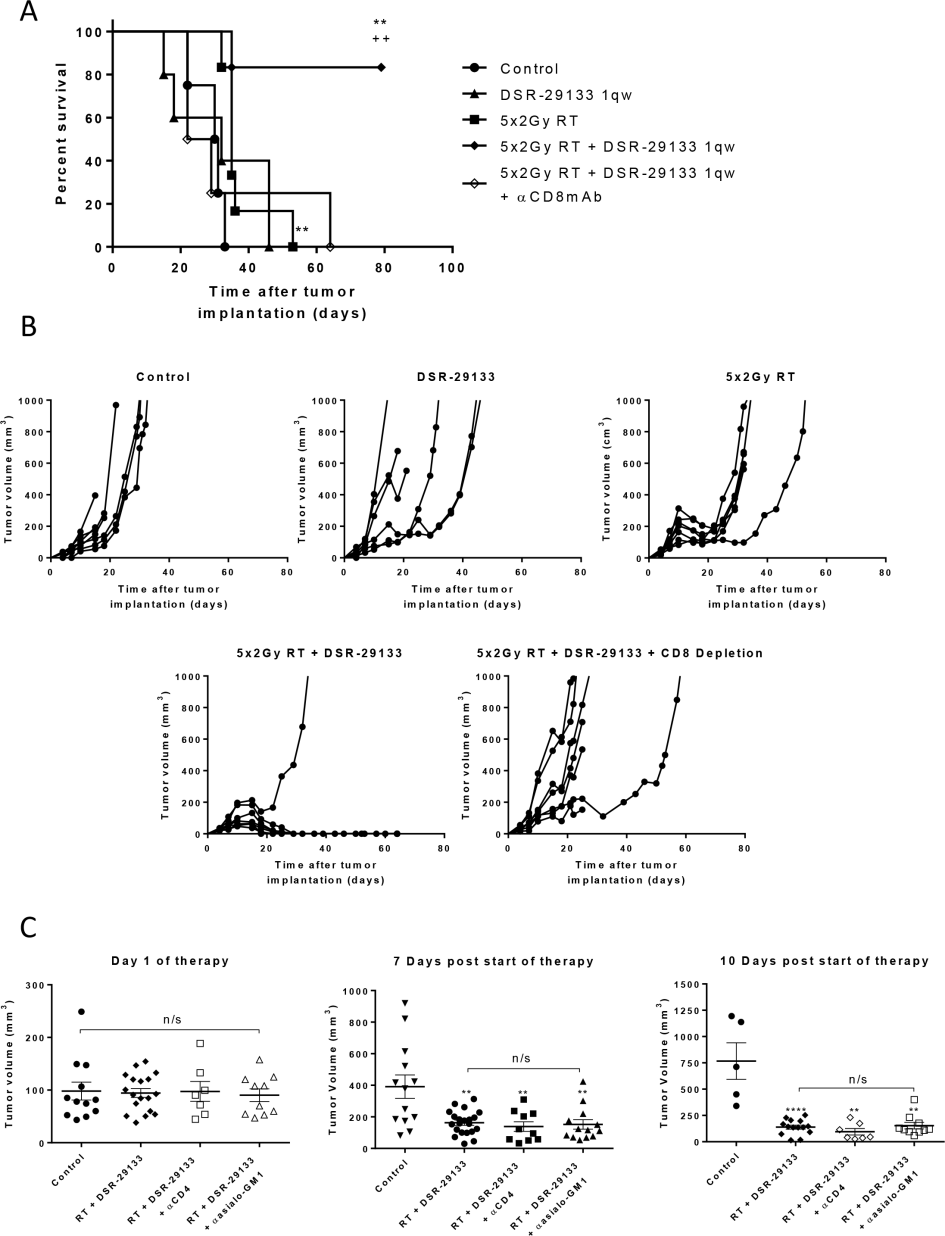
Therapeutic efficacy of fractionated RT and DSR-29133 combination is dependent on the activity of CD8^+^ T-lymphocytes (**A** and **B**) Kaplan-Meier curve (A) and tumor growth (B) of therapy. CD8^+^ T-cells were depleted 1 day prior to therapy with depletion maintained for 2 weeks. (**C**) Tumor volume on day of therapy and 10 days post combination therapy with 5 fractions of 2 Gy and DSR-29133. CD4 or NK-cells were depleted 1 day prior to therapy with depletion maintained for 2 weeks. (A), **/++ *P* < 0.01, Log-rank (Mantel-Cox) test. (C), n/s, *P* < 0.05, Mann-Whitney test. Data representative of at least 5 mice per cohort. *denotes significance when compared to control mice. +, denotes significance when compared to monotherapy.

Using cellular depletion studies we next determined the relative contribution of CD4^+^, CD8^+^ lymphocytes and NK/NKT-cells to tumor control following combined DSR-29133/RT therapy in the CT26 model. In contrast to the efficacy observed with either monotherapy alone, combined therapy led to curative responses in up to 80% of treated mice. The efficacy of this combination was completely dependent on the activity of CD8^+^ T-cells (Figure 4A and 4B). Moreover, the depletion of CD8^+^ T-cells in mice that received combination DSR-29133 and RT treatment reduced median survival to 25.5 days, lower than that observed from cohorts treated with RT alone (35 days). These data suggest that the efficacy provided by RT when delivered as a monotherapy is, at least in part, dependent on the activity of CD8^+^ T-cells in this model. In contrast, the depletion of either CD4^+^ T-cells or NK/NKT cells did not significantly impact therapeutic response (Figure 4C).

**Figure 5:**
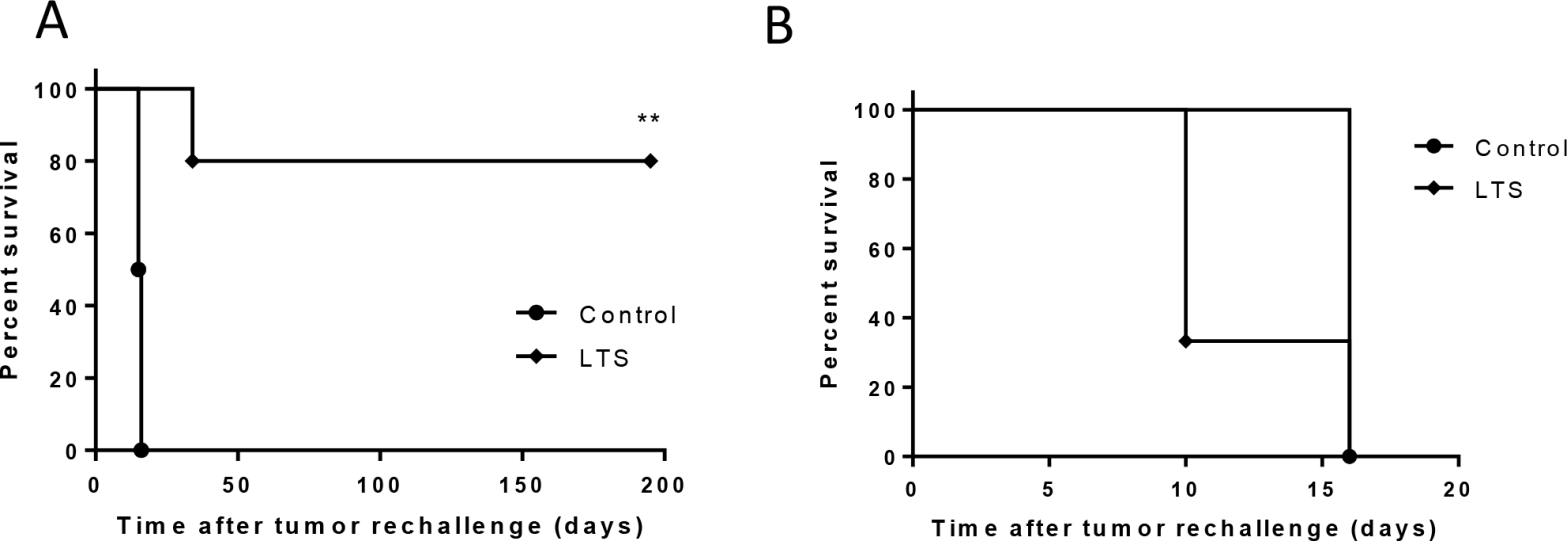
DSR-29133 when combined with fractionated RT generates protective immunological memory (**A** and **B**) Kaplan-Meier curves of tumor naïve and long-term surviving (LTS) mice originally treated with DSR-29133 and RT, following contralateral rechallenge with either 5 × 10^5^ CT26 cells (A) or 1 × 10^5^ 4T1 cells (B). **P < 0.01 compared to control mice (log-rank; Mantel-Cox test). Experimental groups contained at least 4 mice and are representative of at least 2 independent experiments.

Long-term surviving (LTS) mice that underwent curative responses following combined DSR-29133/RT therapy were rechallenged with CT26 tumor cells to determine whether immunological memory had been engendered. We show that at least 75% of LTS mice were capable of suppressing tumor growth following rechallenge (in contrast to 0% in tumor naïve control mice) (Figure 5A). As we had previously demonstrated that combination therapy with DSR-29133/RT generated CD8^+^ T-cell responses capable of recognizing 4T1 cells during ex-vivo co-culture (Figure 3B and 3C) we rechallenged LTS mice with 4T1 cells. Interestingly, whilst LTS mice were protected against rechallenge with CT26 no protection was afforded against rechallenge with 4T1 cells suggesting that memory responses to gp70 alone are not sufficient to mediate tumor control in this setting.

Taken together these data demonstrate that RT when combined with DSR-29133 can generate protective immunological memory against the parental tumor in long-term survivors.

### Concurrent treatment with DSR-29133 administered on day 1 of the fractionated RT cycle is required for improved anti-tumor activity

**Figure 6:**
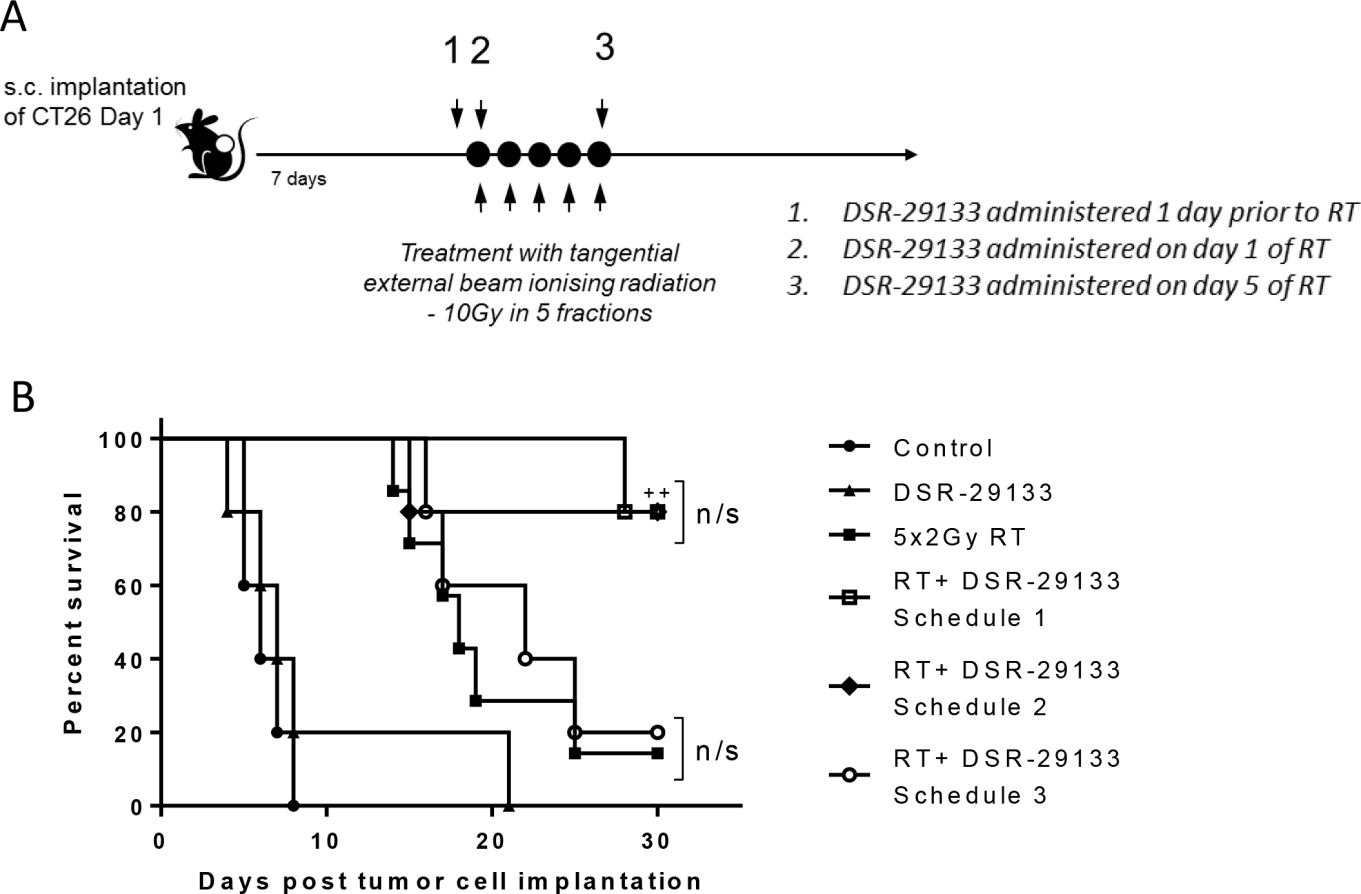
RT potentiation requires administration of DSR-29133 on day 1 of the fractionated RT cycle (**A**) Schema for dose-scheduling studies. Mice received fractionated-dose RT (as 10 Gy in 5 daily fractions of 2 Gy) alone or in combination with DSR-29133 starting either 24 hours prior to the start of RT (schedule A), on day 1 of RT cycle (schedule **B**) or on day 5 of the RT cycle (schedule **C**). (B) Kaplan-Meier curves of therapy. ++, *P* < 0.01 compared to monotherapy (log-rank; Mantel-Cox test). Experimental groups contained at least 7 mice and are representative of 2 independent experiments.

The impact of dose-scheduling of a systemically administered TLR7 agonist and RT on the generation of therapeutic anti-tumor immune responses is currently unclear. We evaluated 3 combination schedules where a fractionated RT cycle of 10 Gy in 5 fractions was administered in combination with DSR-29133 commencing either 1 day before the fractionated RT cycle (schedule 1), day 1 of the cycle (schedule 2) or day 5 of the cycle (schedule 3) (Figure 6A). No significant difference in survival was found between schedule 1 and 2 with survival at 80% in both groups at the study endpoint (*P >* 0.05 Log-rank; Mantel-Cox test) (Figure 6B). In contrast, when DSR-29133 treatment was delayed until the end of the fractionated RT cycle (schedule 3), the combination was completely ineffective at enhancing overall survival when compared to RT alone (median survival of 22 days vs. 18 days respectively; *P >* 0.05 Log-rank; Mantel-Cox test). These data demonstrate that scheduling of DSR-29133 with fractionated RT can impact on the generation of therapeutic CD8^+^ T-cell responses and have important implications for clinical trial design.

## DISCUSSION

Here we demonstrate the therapeutic potential of DSR-29133, a novel systemically administered, low molecular weight adenine derivative agonist of TLR7. To date, many of the reported TLR7 agonists are imidazoquinoline derivatives which also have some activity towards TLR8 [17]. In contrast, we show that DSR-29133 is highly potent and selective for TLR7. PK profiling reveals that DSR-29133 is favorable for i.v. administration due to rapid clearance after administration which may reduce some of the tolerability issues associated with i.v. dosing of imidazoquinoline derivatives. We demonstrate that DSR-29133 is well tolerated in the mouse following i.v. administration, leads to the activation of effector immune cell populations and has single agent anti-tumor activity in multiple syngeneic tumor models. As a monotherapy, 1qw dosing of DSR-29133 significantly reduced tumor burden in both solid tumor and disseminated disease settings however complete responses were only observed when administered in combination with low-dose fractionated RT. Our data reveal that combined therapy generates tumor antigen-specific CD8^+^ T-cell responses which are essential for therapeutic response. LTS mice are protected against tumor rechallenge through the generation of immunological memory. Moreover, we show that correct dose scheduling is critical for the generation of effective anti-tumor immunity.

Topically administered TLR7 agonists such as imiquimod (Aldara, Graceway Pharmaceuticals) have demonstrated clinical effectiveness in dermatological malignancy. More recently, topical (intravesical) administration of TMX-101 (Vesimune, Telormedix) was granted orphan drug designation by the food and drug administration (FDA) for the treatment of non-muscle-invasive bladder cancer (NMIBC) [18]. However, for many other cancer types, systemic or intra-tumoral delivery of TLR7 agonists may be required to elicit anti-tumor immune responses. Recent preclinical and clinical studies have demonstrated the immunogenicity of TLR agonists when delivered intra-tumorally [19, 20]. However, direct intratumoral administration to non-cutaneous tumors requires image-guided delivery and may be challenging where repeated administration is required. I.v. administration would obviate these issues and permit systemic activation of innate and adaptive effector immune cells.

It is becoming evident that combination therapies are required to exploit maximal anti-cancer immunity. In addition to targeting additional co-inhibitory and co-stimulatory pathways, combination with standards of care or targeted therapies that lead to tumor cell stress and/or death may improve the immunogenicity of TLR7 therapy. In the present study our data reveal that despite the capacity of i.v. DSR-29133 administration to engender systemic immune activation, as a monotherapy this was unable to mediate complete tumor resolution. Combining with RT may provide an ‘*in situ* vaccination’ effect leading to increased availability of tumor associated antigens (TAAs), provision of DAMPs and to modulation of the tumor microenvironment to favor DC recruitment, activation and generation of T-cell responses [9–14]. However, preclinical data demonstrate that in the setting of established disease treatment with RT alone is insufficient to generate therapeutic immune responses capable of long-term disease control. This is mirrored in the clinical setting where disease recurrence is the leading cause of mortality following RT. We and others have demonstrated the potential to improve systemic anti-tumor immune responses through combination of RT with immunotherapy [6–8, 21].

In the present study, we demonstrate that the combination of DSR-29133 and fractionated RT leads to the generation of tumor antigen specific CD8^+^ T-cell responses in mice bearing established CT26 tumors. We show that following combined therapy there is an expansion in the frequency of CD8^+^ T-cells specific for the H2-Ld restricted epitope, AH1 (a peptide derived from an endogenous retroviral gene product; gp70) which is highly expressed on CT26 tumor cells [22, 23]. The frequency of CD8^+^ T-cells capable of recognizing CT26 cells following ex-vivo co-culture with irradiated CT26 cells is > 2 fold higher than following AH1 peptide co-culture demonstrating priming against additional antigenic epitopes. Increased CD8^+^ T-cell responses were also observed following ex-vivo co-culture with 4T1 cells, which also express the gp70 antigen. Subsequent rechallenge studies revealed that LTS mice (CT26 tumor bearing and originally treated with DSR-29133/RT) were protected against tumor rechallenge following inoculation with CT26 but not 4T1 tumor cells. This is despite the generation of AH1-specific CD8^+^ T-cells in mice following combined therapy that are capable of recognizing 4T1 cells. Together these data suggest that responses against gp70 epitopes alone may be insufficient to provide effective tumor control and that CD8^+^ T-cell recognition of additional TAA's are required for tumor control of the 4T1 tumor. These observations are supported by recent clinical studies that reveal the polyclonal nature of anti-tumor T-cell responses in patients [24] and the increased T-cell receptor diversity observed following high-dose RT in murine models [25].

A recent study by Schölch et al. demonstrated that intratumoral administration of the TLR7 agonist 3M-011 stimulated both NK and CD8^+^ T-cell dependent anti-tumor responses [26]. However, only CD8^+^ T-cells contributed to long-term therapeutic efficacy when 3M-011 was administered in combination with RT, mirroring the responses observed in the present study. Previous studies have demonstrated the capacity of RT to induce APC-mediated cross-priming of T-cells that contribute to the therapeutic response [21]. Taken together these data suggest that RT can modulate the phenotype of response generated by TLR7 agonist-mediated immune activation possibly through the provision and uptake of TAAs by APC, subsequent to RT-induced tumor cell death.

We have previously demonstrated that sequencing of RT with immune checkpoint inhibitors can substantially impact therapeutic response [7]. To date, no studies have addressed the impact of sequencing on the efficacy of RT and systemic TLR7 agonist therapy. We show that administration of DSR-29133 either 24 hours prior to, or on day 1 of the fractionated RT cycle can enhance therapeutic response. In contrast, when treatment with DSR-29133 is initiated on the final day of the fractionated RT cycle the therapeutic response is equivalent to RT alone. It is possible that the administration of TLR7 agonist needs to be synchronous with the RT-induced changes in the tumor microenvironment such as tumor cell death and release of TAAs and DAMPs to augment successful CD8^+^ priming. However, further studies are required to determine why concurrent therapy is required. These data reveal the importance of scheduling immunotherapy and RT combinations for successful potentiation of therapeutic response and have direct relevance to clinical trial design.

In summary, this study describes the novel TLR7 agonist DSR-29133 which is well tolerated when administered systemically and leads to acute immune activation and anti-tumor activity when administered as a monotherapy. Moreover, we show that DSR-29133 can be successfully combined with fractionated RT and when delivered concurrently can elicit CD8^+^ T-cell dependent efficacy and generate memory immune responses that protect against disease recurrence. DSR-29133 is a promising TLR7-selective agonist for the treatment of solid malignancies and translation to early phase clinical trial is clearly warranted.

## MATERIALS AND METHODS

### Materials

DSR-29133 was synthesized by Chemistry Research Laboratories, Drug Research Division, Sumitomo Dainippon Pharma Co., Ltd.

### Cell culture

NF-kB/SEAP/293 cells (Imgenex, Japan) were maintained in DMEM supplemented with 10% fetal bovine serum, 500 μg/mL geneticin, 100 U/mL penicillin and 100 U/mL streptomycin (Invitrogen, Japan). The murine osteosarcoma cell line, LM8 (RIKEN Bioresource Center, Japan) was maintained in RPMI 1640 supplemented with 10% fetal bovine serum, 2 mM L-glutamine, 100 U/mL penicillin and 100 U/mL streptomycin (Invitrogen). The murine renal cell carcinoma cell line, Renca (kindly provided by Dr. T. Fujioka, Iwate Medical University School of Medicine, Iwate, Japan) was maintained in RPMI-1640 medium supplemented with 10% fetal bovine serum, 2 mM L-glutamine, 100 U/mL penicillin and 100 U/mL streptomycin (Invitrogen). The murine colorectal adenocarcinoma cell line, CT26 (American Type Culture Collection) was maintained in DMEM supplemented with 10% foetal bovine serum and 2 mM L-glutamine (both from Invitrogen, U.K.). The triple negative breast cancer cell line 4T1 (LGC Standards, U.K.) was maintained in RPMI-1640, supplemented with 10% FCS, 1% L-glutamine (Invitrogen, U.K.). All cell lines were cultured to limited passage prior to implantation and were routinely screened to confirm the absence of Mycoplasma contamination.

### TLR7 reporter gene assay

NF-kB/SEAP/293 cells were stably co-transfected with either human or murine TLR7 (pUNO expression vector) and a pNiFty2-SEAP (secreted alkaline phosphatase) reporter plasmid or with human TLR8 (pCMV-script expression vector) along with the pNFκB-Luc Cis-Reporter plasmid and the SEAP reporter gene under the transcriptional control of an NF- kB response element (IMGENIC). To determine activity and specificity cells were seeded in 96-well then stimulated with varying concentrations of DSR-29133 and incubated for 20 hours at 37°C in 5% CO2. TLR activation was assessed by measuring SEAP activity using the QUANTI-Blue assay (InvivoGen, Japan) according to manufacturer's instructions. Absorbance was measured using a microplate reader (Spectra Max190, Molecular Devices, U.S.A.) at 620 nm.

### Mice

### Pharmacokinetic and pharmacodynamic profiling of DSR-29133 *in vivo*

Dose-finding studies were conducted in Balb/c mice. DSR-29133 was dissolved in 0.0042N HCl saline and adjusted to neutral pH with NaOH prior to administration. Mice received DSR-29133 (0.03–3 mg/kg) and plasma was collected 2 h after dosing. The pharmacokinetic profile of DSR-29133 was measured over 4 hours following a single i.v. injection (1 mg/kg dose). Blood samples were taken into EDTA solution. Following centrifugation at 2,486 × g for 20 minutes at 4°C, plasma samples were collected and mixed with methanol then analyzed in 0.1% formic acid in methanol and 0.1% formic acid in water by LC-MS/MS on a API 4000 LC/MS/MS system (AB SCIEX, U.S.A). For time course studies mice received i.v. DSR-29133 (1 mg/kg) and plasma was collected 0.08–6 h after administration. IP-10 and IL-1Ra concentrations were measured by ELISA (R & D systems) and IFNα activity was measured using a cell based reporter gene bioassay system using L929/2–5AS-Luc cells stably expressing luciferase under a 2′-5′-oligoadenylate synthase promoter (established at Sumitomo Dainippon Pharma). To determine lymphocyte expression of CD69, mice bearing 7 day established CT26 tumors received a single i.v. dose of DSR-29133 (1 mg/kg). 6 hours after dosing, mice were euthanized and CD69 expression on T and B cells determined by flow cytometry following blockade of CD16/32 (Fc (CD16/32): clone 93, CD69: clone H1.2F3, eBiosciences, U.K.; CD4: clone RM4.5, CD8: clone 53.67, BD Pharmingen, U.K and CD19: 1D3, eBiosciences, U.K.) using an LSRII (Becton Dickinson, U.K.) and analyzed using FlowJo (Tree Star, U.S.A.).

### Tumor studies

For the metastatic osteosarcoma line LM8, C3H mice were inoculated subcutaneously (s.c.) on day 0 with 3.3 × 10^6^ cells in Hank's Balanced Salt Solution (HBSS). DSR-29133 was dissolved in 0.0042N HCl saline and adjusted to neutral pH with NaOH before administration. Mice were administered either DSR-29133 (0.1 mg/kg) or vehicle by intravenous (i.v.) injection. Treatment was initiated on day 7 after implantation and repeated once weekly, up to a maximum of four treatments. Tumor growth was measured twice weekly and mice were euthanized 28 days after primary tumor inoculation to determine the extent of lung metastasis. Briefly, lungs were fixed in 4% buffered formalin and metastasis scored on haematoxylin and eosin-stained sections by microscopy.

Balb/c mice were inoculated s.c. on day 0 with either Renca or CT26 tumor cells (1 × 10^5^ or 4 × 10^4^ cells in HBSS, respectively). Mice received either 0.1 or 1 mg/kg DSR-29133 (as described above) with treatment commencing on day 1 (Renca) or day 7 (CT26) after implantation, for up to a maximum of 5 treatments. Irradiation of CT26 tumors commenced 7 days after inoculation (when tumors were approximately 100 mm^3^) using a Pantak HF-320 320 kV x-ray unit (Gulmay Medical, U.K.). The machine was operated at 300 kV, 9.2 mA, with filtration fitted in the x-ray beam to give a radiation quality of 2.3 mm Cu half-value layer. Mice were positioned at a distance of 350 mm from the x-ray focus, where the dose rate was 0.80 Gy/min.

For cellular depletion experiments mice received either αCD8 mAb clone YTS169 (a gift from M. Glennie, Southampton University); αCD4 mAb clone GK1.5 (Biolegend) or αAsialo-GM1 (Wako Chemicals). Peripheral blood was sampled during therapy to confirm cellular depletion. For tumor rechallenge experiments, long-term surviving (LTS) mice were implanted contra-laterally with tumor cells a minimum of 80 days after previous tumor implantation.

For all tumor studies, animals were monitored daily and weighed at least twice weekly. Tumors were measured twice weekly with calipers, and tumor size was calculated as the product of the perpendicular diameters of individual tumors. Tumor volume (1000 mm^3^) was the primary endpoint for efficacy studies. Experimental groups contained at least 5 mice/group and are representative of at least 2 independent experiments. *In vivo* experiments were conducted either according to a U.K. Home Office license or according to the guidelines of the Animal Care and Use Committee at Sumitomo Dainippon Pharma Co., Ltd. Japan.

### Measurement of cytokine production by CD8^+^ T-cells isolated from mice post therapy

Splenocytes were isolated from either control mice or DSR-29133/RT-treated mice 10 days after the start of therapy and co-cultured with either irradiated tumor cells (50 Gy) or 1 μmol/ml of the H2-Ld restricted peptides SPSYVYHQF (AH1)/TPHPARIGL (β-galactosidase) (Anaspec, U.K.) as described previously (22). Experimental groups contained 3–5 mice and are representative of 2 independent experiments.

### Statistical analysis

*In vivo* data were analyzed with non-parametric Mann-Whitney test or Wilcoxon matched-pairs signed rank test. *In vitro* data was analyzed with two-tailed unpaired Student's t test unless stated otherwise. For all statistical tests, statistical significance accepted when *P* < 0.05. Data expressed as mean ± SEM unless otherwise indicated.

## SUPPLEMENTARY MATERIALS FIGURES


