# Supplementary material for: Intravenous administration of the selective toll-like receptor 7 agonist DSR-29133 leads to anti-tumor efficacy in murine solid tumor models which can be potentiated by combination with fractionated radiotherapy
Source: Oncotarget. 2016 Mar 5;7(13):17035–46. doi: 10.18632/oncotarget.7928 (PMC4941369; doi:10.18632/oncotarget.7928)
Supplement: Supplementary file 1 [file oncotarget-07-17035-s001.pdf]

Intravenous administration of the selective toll-like receptor 7 agonist DSR-29133 leads to anti-tumor efficacy in murine solid tumor models which can be potentiated by combination with fractionated radiotherapy

Supplementary Materials

SUPPLEMENTARY METHODS

Colony forming assays

Exponentially growing CT26 cells were plated at densities of 1×10^2 to 2.4×10^3 well. 24 h later, cells were treated with a range of concentrations between 0 and 10 µM DSR-29133 followed 2 h later by X-ray radiation treatment with 0, 2 or 4 Gy. Media were replaced 24 h later, or left unchanged, and cells were left to proliferate for 7 days or until colonies of ~50 cells were present in the untreated wells. Colonies were stained with 0.5 % (w/v) methylene blue solution and counted manually. Surviving fractions were normalised to the non-irradiated control or in the case of experiments not involving radiation, the untreated control.

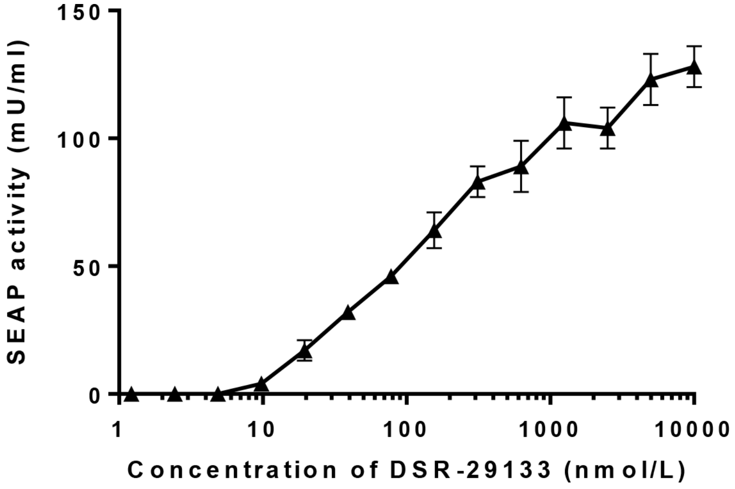

Supplementary Figure S1: DSR-29133 is active against the mouse TLR7 receptor. A, NFκB gene reporter assays were performed in HEK293 cells transfected with either murine TLR7 (0.001–10 µM) of DSR-29133. Data expressed as mean ± SEM Data are representative of at least 2 independent experiments.

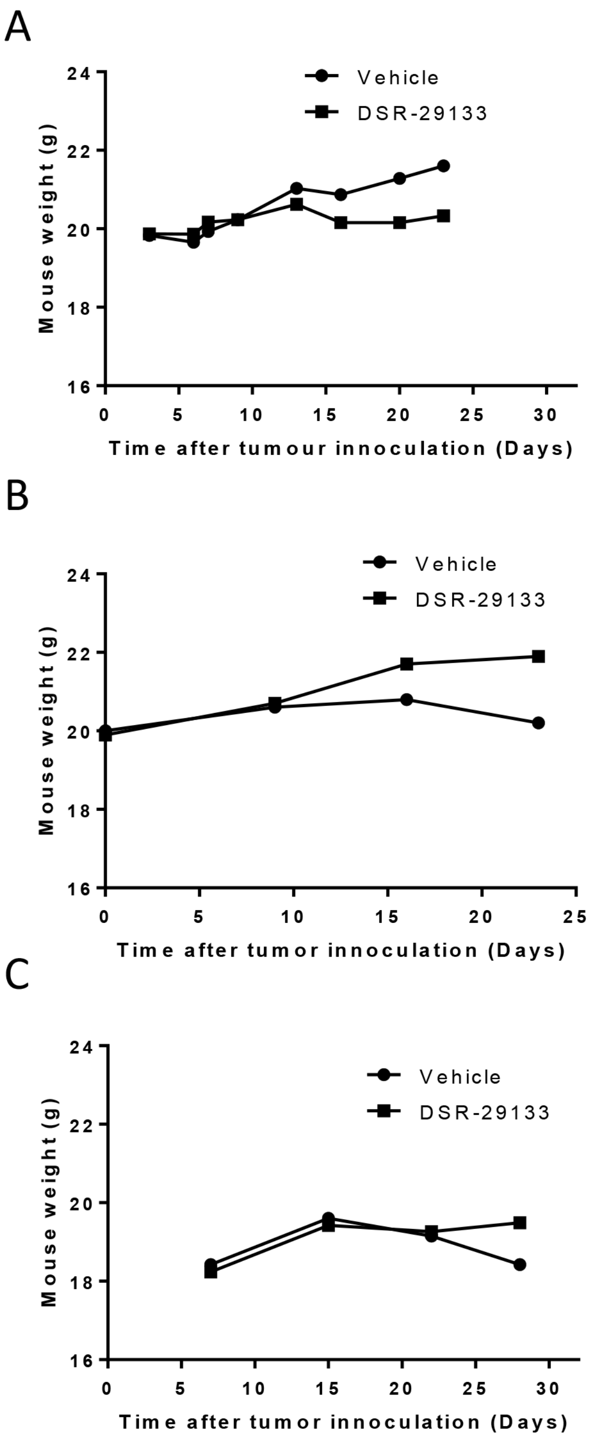

Supplementary Figure S2: DSR-29133 is well tolerated following intravenous administration. (**A**) CT26, (**B**) Renca (both Balb/c) and (**C**) LM8 (C3H) tumor bearing mice received weekly i.v. injections of DSR-29133 at a dose of 0.1 (Renca and LM8) or 1 mg/kg (CT26) for 3 weeks. Individual mouse weight plotted.

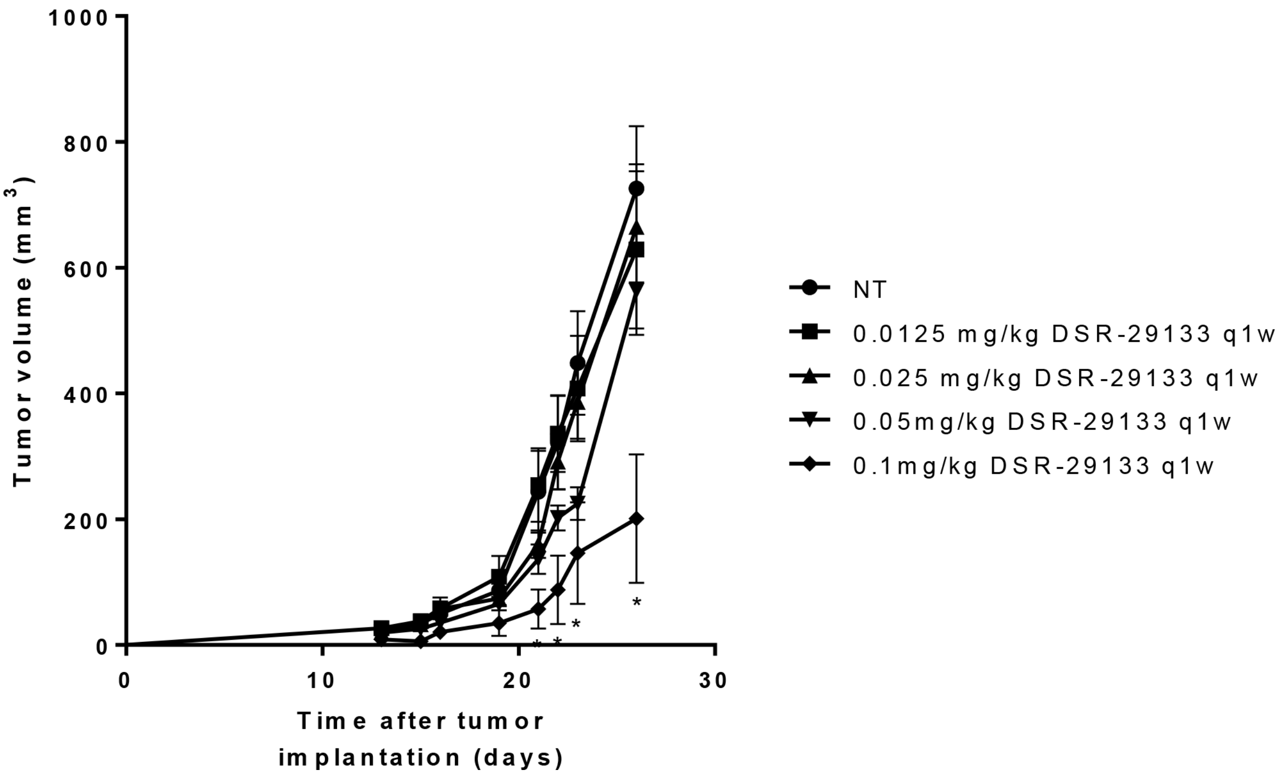

Supplementary Figure S3: DSR-29133 leads to a dose-dependent reduction in Renca tumor volume following intravenous administration. Renca tumor bearing (Balb/c) mice received weekly i.v. injections of DSR-29133 at doses of 0.0125–0.1 mg/kg for 3 weeks. Data expressed as mean ± SEM. Experimental groups contained at least 5 mice. *$P < 0.05$ (Mann-Whitney test) when compared to NT cohort.

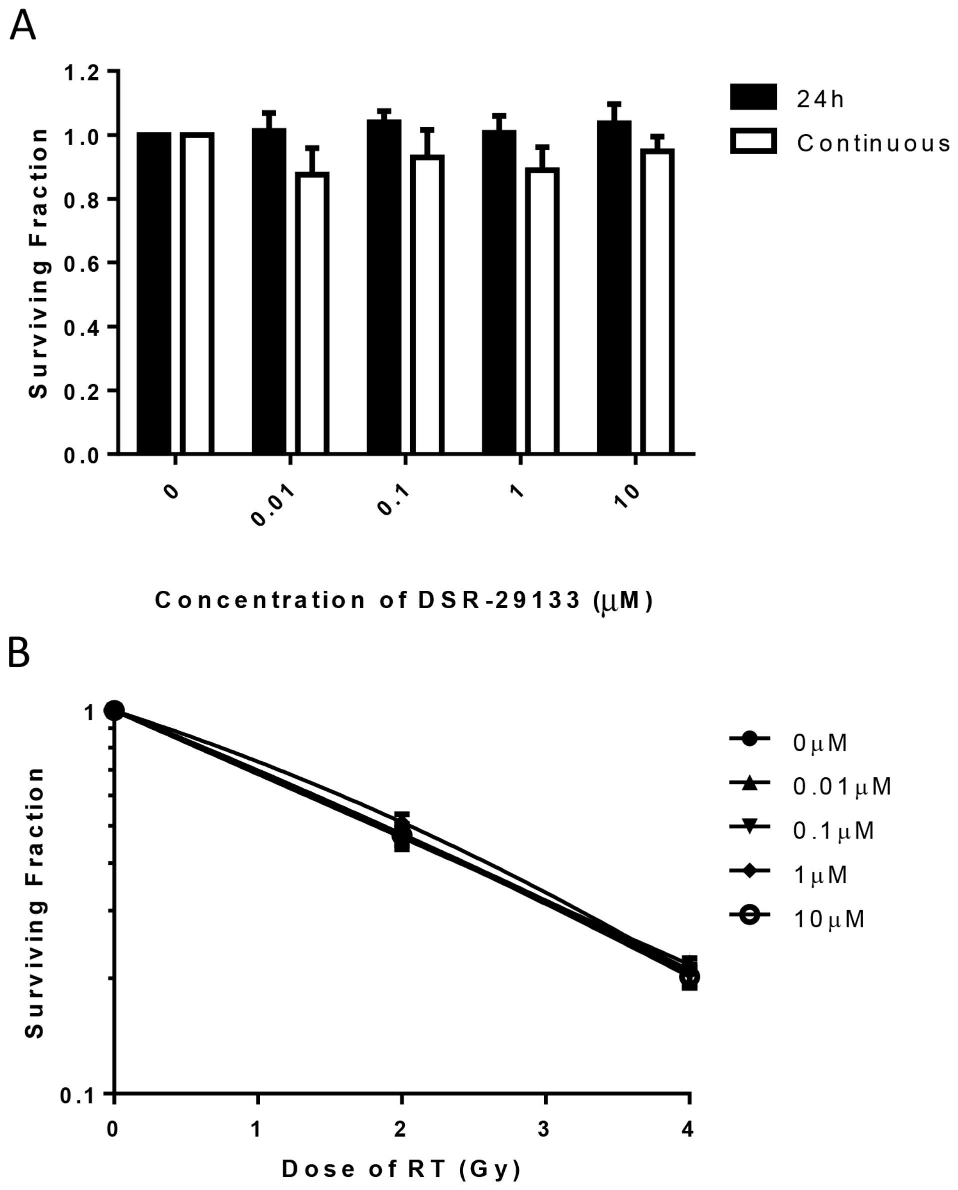

Supplementary Figure S4: DSR-29133 does not alter tumor cell clonogenic potential or radiosensitivity of CT26 cells. (**A**) Colony forming assays using 0.01–10μM DSR-29133 does not affect tumor cell clonogenicity when added to the culture medium either for 24 hours or for the duration of the experiment. (**B**) Addition of DSR-29133 does not impact the radiosensitivity of CT26 cells. Data expressed as mean ± SEM Data are representative of at least 2 independent experiments.
